# Supplementary material for: EGCG-Mediated Protection of Transthyretin Amyloidosis by Stabilizing Transthyretin Tetramers and Disrupting Transthyretin Aggregates
Source: Int J Mol Sci. 2023 Sep 15;24(18):14146. doi: 10.3390/ijms241814146 (PMC10531593; doi:10.3390/ijms241814146)
Supplement: Supplementary file 1 [file ijms-24-14146-s001.zip › ijms-2571743-supplementary.pdf]

EGCG-mediated Protection of Transthyretin Amyloidosis by Stabilizing Transthyretin Tetramer and Disrupting Transthyretin Aggregates

Huizhen Zou[1], Shuangyan Zhou*

[1] Chongqing Key Laboratory of Big Data for Bio Intelligence, Chongqing University of Posts and Telecommunications, Chongqing 400065, China
* Corresponding author: zhousy@cqupt.edu.cn

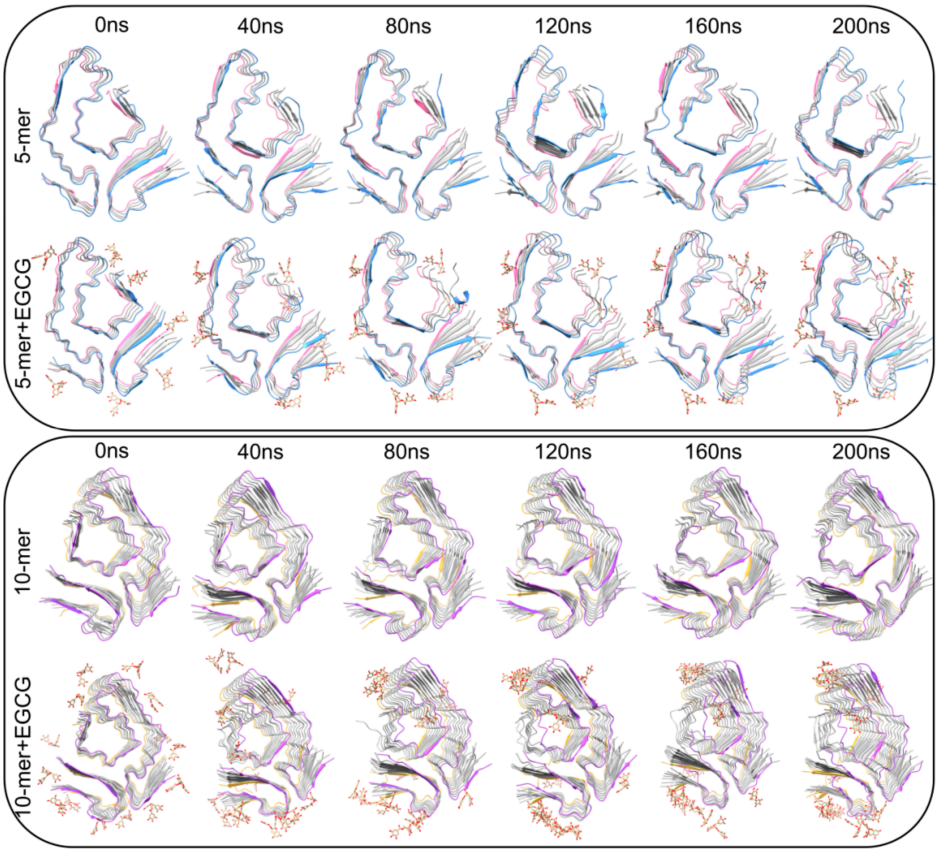

Figure S1. Structural presentation of TTR aggregates during simulation with an interval of 40 ns.

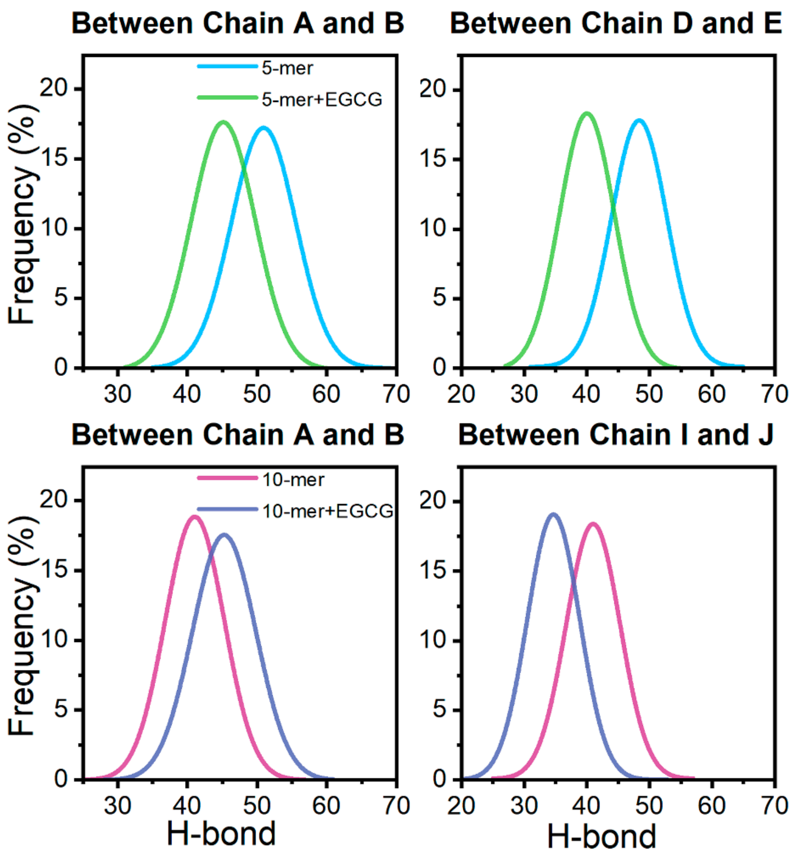

Figure S2. Distribution of backbone hydrogen bonding between boundary β-strands and adjacent β-strands.

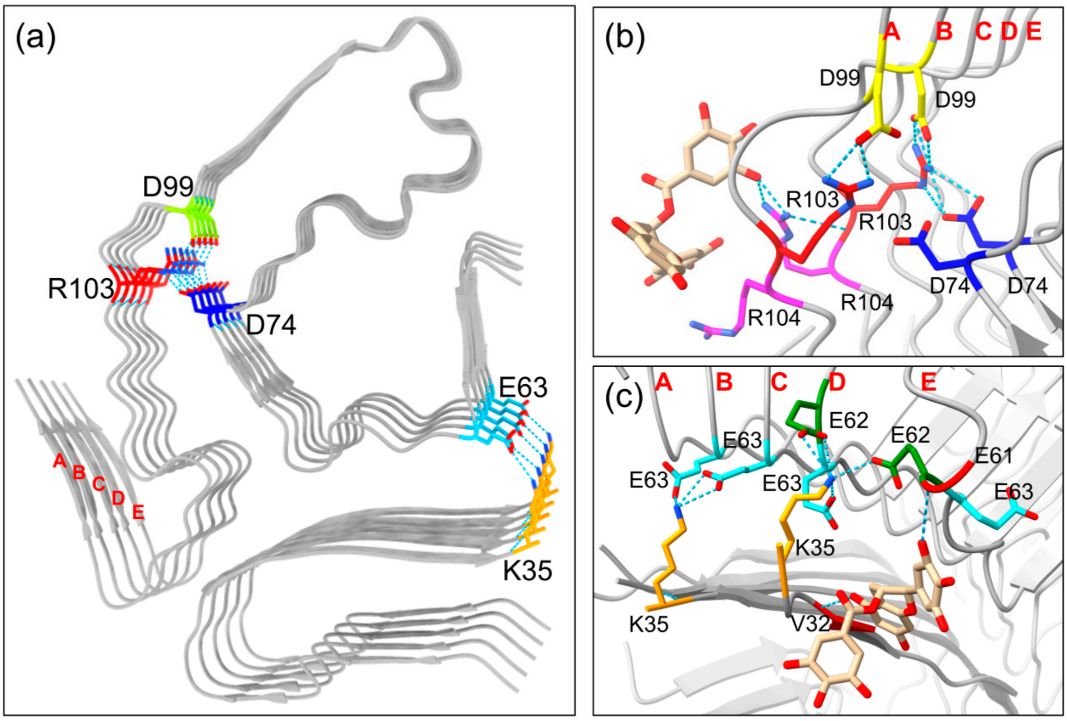

Figure S3. (a) Graphic of the experimental model with key interactions labeled; (b) and (c) interactions of EGCG with residues at site 1 and site 2, respectively. Letters A-D in each picture represent for the β-strand chain of TTR pentamer.

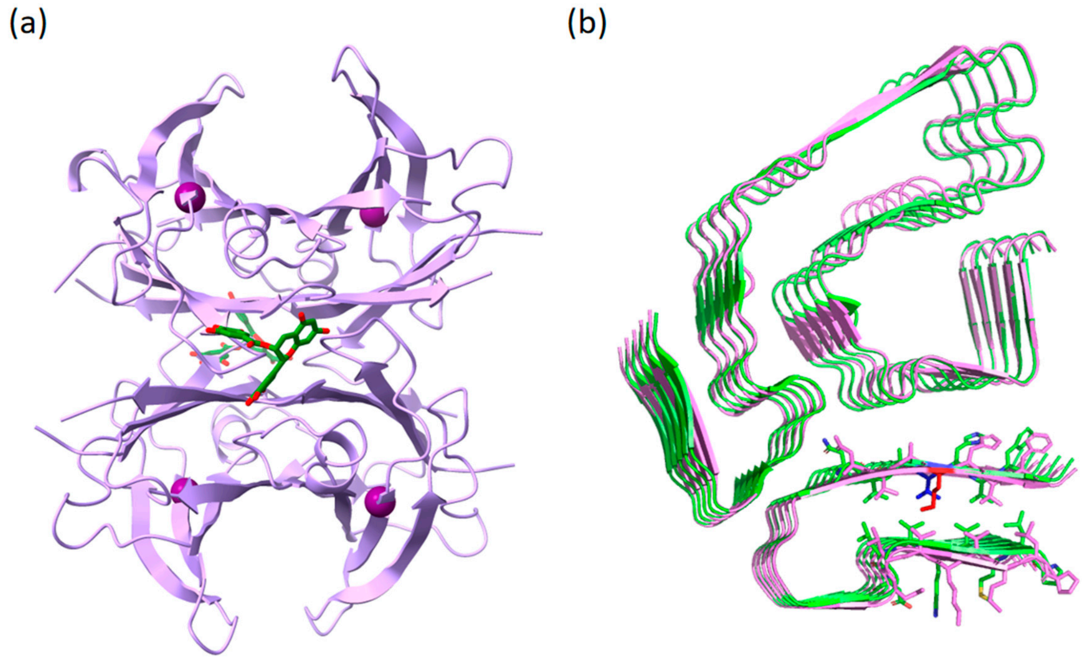

Figure S4. (a) Structure of V30M TTR tetramer with EGCG binding at site 1, purple balls represent for the location of residue 30 in each chain; (**b**) Superposition of WT and V30M TTR aggregates, pink is for V30M mutant, green is for WT, red stick indicates M30, and blue indicates V30.

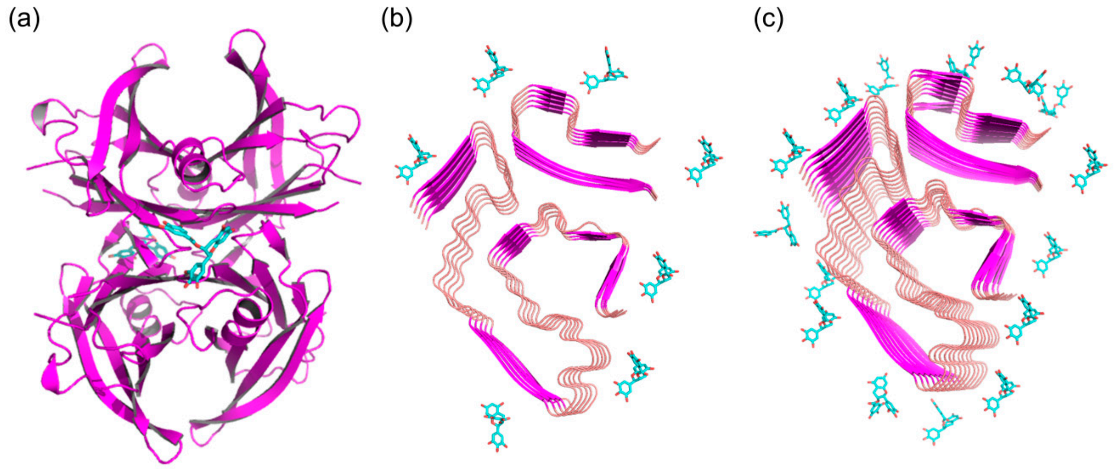

Figure S5. Initial structure of simulated structure. (**a**) Binding of EGCG to TTR tetramer at site 1; (**b**) initial structure of 5-mer with 7 EGCG placed randomly; (**c**) initial structure of 10-mer with EGCG placed randomly. TTR is depicted in purple, and EGCG is depicted in blue. And for both initial structure of 5-mer and 10-mer with EGCG, there is no hydrogen bonding interaction between EGCG and 5-mer, 10-mer.
